# Analysis of Attitudes Toward Breastfeeding and Spiritual Life During Pregnancy in Türkiye: A Qualitative Study

**DOI:** 10.1007/s10943-024-02235-w

**Published:** 2025-02-13

**Authors:** Yeliz Dinçer, Şeyma Kilci Erciyas

**Affiliations:** 1 Department of Nursing, Faculty of Health Sciences, Zonguldak Bulent Ecevit University, Zonguldak, Turkey; 2 Department of Nursing, Faculty of Health Sciences, Amasya University, Amasya, Turkey

**Keywords:** Pregnancy, Qualitative research, Religion, Spirituality

## Abstract

It is crucial for nurses to understand the meaning of spirituality, which can become more pronounced during pregnancy, childbirth, and breastfeeding, and to take a supportive approach to breastfeeding attitudes during pregnancy. This study aimed to explore pregnant women’s attitudes and spiritual experiences in Türkiye regarding breastfeeding. A qualitative inductive content analysis design was employed. Seventeen pregnant women (36–39-week gestation) were admitted to a maternity hospital in Zonguldak, Türkiye. They were selected using purposive sampling. Data were collected through semi-structured, face-to-face, in-depth interviews and analyzed using an inductive content analysis approach. The mean age of the participants was 27.41 years, and ten participants were primiparous. Multiparous participants had previously breastfed for a mean duration of 19.1 months. Three main themes emerged: “Breastfeeding and life,” “Spiritual life and breastfeeding,” and “Cultural synthesis.” Considering the impact of spirituality on breastfeeding during pregnancy, these findings may help nurses recognize pregnant women’s spiritual needs and value the protective role of spirituality. Understanding these dimensions could improve the quality of support provided by healthcare professionals and potentially enhance breastfeeding outcomes.

## Introduction

Breastfeeding is influenced by a wide range of physical, mental, social, and cultural factors. The initiation and maintenance of breastfeeding are closely related to the mother’s health and well-being during the prenatal period (de Argolo Cerqueira et al., 2018). Attitudes toward breastfeeding are shaped by personal beliefs, social pressures, and cultural norms (Yanıkkerem et al., 2014). Factors such as socioeconomic status, social life, employment conditions, lack of knowledge, fear, and cultural trends all play a role in determining whether a mother chooses to breastfeed (Akçay et al., 2021; Yanıkkerem et al., 2014; Yiğitbaş et al., 2012).

Lactation is one of the key physiological stages of motherhood. The female breast can be viewed in multiple ways: as a nourisher of life, a symbol of sanctity, or, conversely, as a sexual object. Associations with chastity, privacy, and social norms have made breastfeeding a sensitive subject. Respect for maternal privacy is important worldwide and is particularly emphasized among Muslim mothers (Farhadi, 2020; Timurkan, 2020). Religious practices can shape knowledge, attitudes, and behaviors related to infant feeding. Healthcare professionals should be aware of the religious and spiritual factors that may influence breastfeeding. Spiritual aspects that can serve as motivators include the belief that breast milk has sacred qualities, as well as prayer and support from religious communities (Adepoju et al., 2019; Hirani & Ratushniak, 2022).

Spirituality, representing an individual’s search for meaning, purpose, and interconnectedness, often becomes more important during sensitive periods such as pregnancy (Doğru, 2022; Heidari et al., 2015; Karataş Temiz, 2019). While spirituality was once considered synonymous with religion, it now encompasses a broader range of experiences, values, and beliefs (Çınar & Eti Aslan, 2017; Dhamani, 2014). Meeting spiritual needs is crucial during pregnancy (Adib-Hajbaghery et al., 2017). Religion and spirituality can help individuals find meaning in challenging life events (Mann et al., 2010; Price et al., 2007). Therefore, cultural and religious traditions may support health-promoting behaviors, including breastfeeding (Redelinghuys et al., 2014). Although some attention has been paid to religious or spiritual aspects of breastfeeding, these factors have not been extensively explored. Understanding spirituality’s influence on breastfeeding may help nurses and other healthcare professionals provide more holistic and culturally sensitive care. This study aims to evaluate breastfeeding from a spiritual perspective among pregnant women in Türkiye, contributing new insights to the literature.

## Methods

This qualitative descriptive study was conducted in Türkiye between October 2022 and May 2023, using an inductive content analysis approach to understand pregnant women’s attitudes toward breastfeeding and their spiritual life experiences.

### The Universe and Sample of the Research

Within the scope of the study, 17 primiparous and multiparous pregnant women were interviewed.

### Criteria for İnclusion in the Research

- Having no health problems,
- Not being in active labor,
- Being in pregnancies over 36 weeks of gestation

### Exclusion Criteria for the Research

- Risky pregnancies,
- Pregnancies under 36 weeks,
- Those in active labor

The study was completed, and the sample size was terminated when the saturation point was reached. The phenomenological approach, a qualitative research pattern, aims to define the common meaning of the experiences of individuals or groups experiencing the phenomenon regarding a phenomenon or concept (Creswell, 2018). Since the research has a qualitative design, the sample size will be determined when it reaches the saturation point. The study was conducted following the consolidated criteria for reporting qualitative research (COREQ) checklist for qualitative research (Tong et al., 2007).

### Data Collection Tools

Data were collected using a “Personal Information Form” and a “Semi-Structured Interview Form Regarding Attitudes Towards Breastfeeding and Spiritual Life,” along with a voice recorder.

#### Personal Information Form

This form was prepared by the researchers by scanning the literature (Akcay et al., 2021; Çınar & Eti Aslan, 2017; Dhamani, 2014; Doğru, 2022; Düzgüner, 2013; Heidari et al., 2015; Karataş Temiz, 2019; Kurnaz & Uyar Hazar, 2021; Timurkan, 2020; Yiğitbaş et al., 2012). This form contained seven questions addressing age, marital status, education level, family structure, gestational week, number of pregnancies, the ages of any living children, and previous breastfeeding duration. Expert opinions were sought from five faculty members specializing in Women’s Health and Disease Nursing and Child Health and Disease Nursing to ensure the accuracy and appropriateness of these questions. After considering their feedback, the final version of the questionnaire was created.

#### Interview Form Regarding Attitudes Toward Breastfeeding and Spiritual Life

This form consisted of 12 open-ended questions designed to gain a deeper understanding of pregnant women’s perspectives on breastfeeding and spiritual life during pregnancy.

### Data Collection Process

Data collection took place through semi-structured, face-to-face, in-depth interviews. Participation was entirely voluntary. Pregnant women who came to the hospital for a non-stress test (NST) procedure were interviewed on the same day in a quiet, private area near the NST room. Each conversation lasted about 30 min. All interviews were recorded with a voice recorder and then transcribed verbatim immediately afterward.

### Analysis of Data

Data were analyzed through a six-stage approach. First, the researcher transcribed the audio recordings and observation notes. The initial stage involved becoming fully familiar with the data by reading the transcripts repeatedly and making notes. Two researchers independently examined these notes, verified the audio transcripts, and reached a consensus on the preliminary coding.

In the second stage, codes were systematically generated from the interview data (Creswell & Poth, 2018). The third stage involved organizing the codes into broader categories. During the fourth stage, the researchers reviewed the emerging themes and associated codes to ensure they fully addressed the study’s research questions. In the fifth stage, the team carefully considered each theme’s naming, scope, and definitions and conducted a detailed analysis. Finally, the researchers compiled and reported the findings in the sixth stage.

## Results

**Fig. 1:**
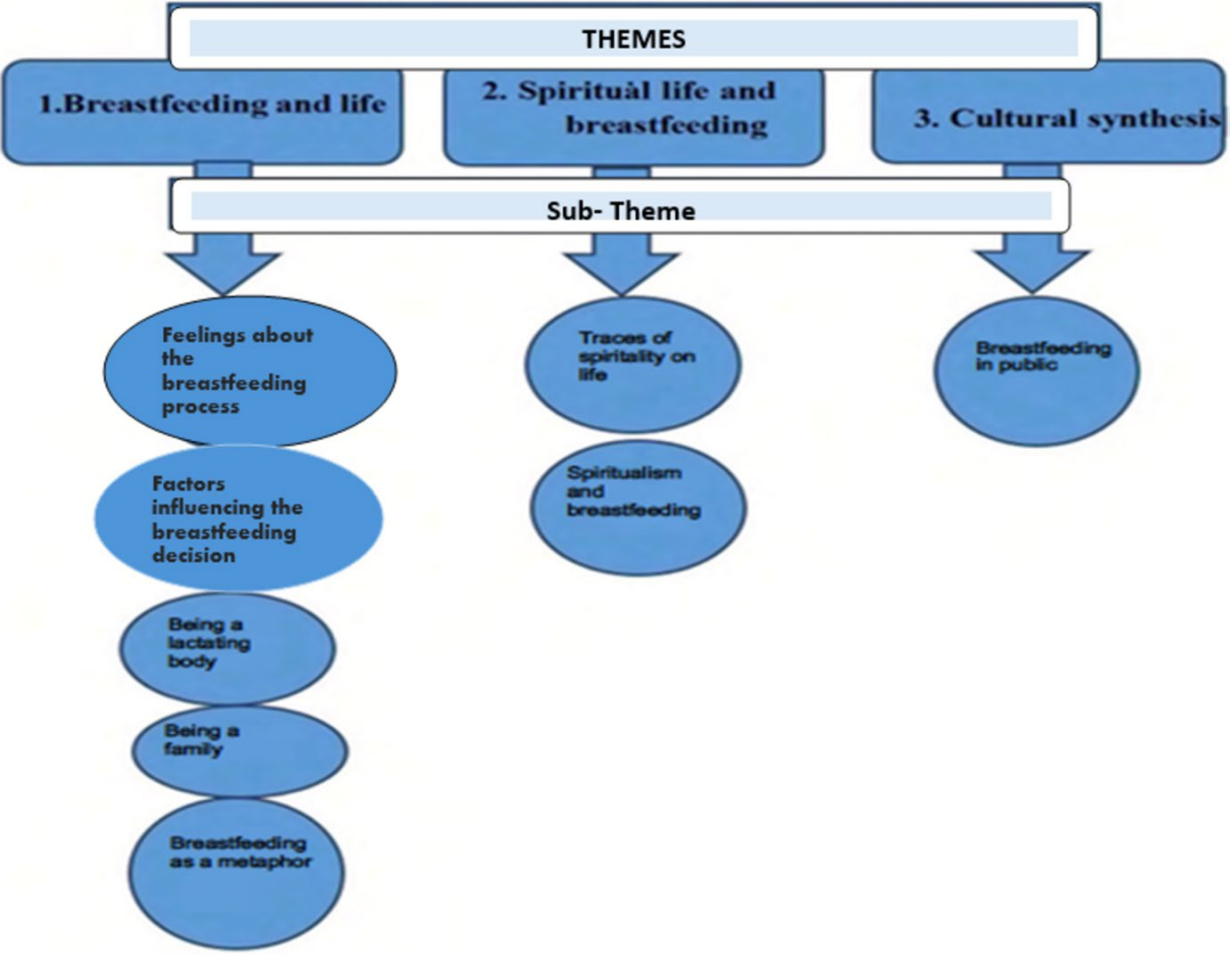
Themes and sub-themes related to the attitudes and spiritual life of pregnant women toward breastfeeding

The socio-demographic characteristics of the participants are presented in Table 1. From the analysis, three main themes emerged: “Breastfeeding and life,” “Spiritual life and breastfeeding,” and “Cultural synthesis.” These overarching themes, along with their sub-themes, are illustrated in Fig. 1.

**Table 1:** Socio-demographical characteristics of pregnant women

No	Age	Education status	Family structure	Gestatıonal age	Number of pregnancies	Previous breastfeeding status/duration
P1	31	University	Nuclear	36	1	–
P2	21	Primary School	Extended	39	1	–
P3	25	University	Nuclear	39	1	–
P4	30	Primary School	Nuclear	39	3	1st child (age 8)/2 years 2nd child (age 4.5)/2 years
P5	24	University	Nuclear	39	1	–
P6	34	University	Nuclear	37	1	–
P7	25	University	Nuclear	37	2	1st child (age 5)/3 years
P8	25	Associate degree	Nuclear	38	1	–
P9	19	Literate	Extended	36	1	–
P10	18	Primary School	Nuclear	38	1	–
P11	33	Associate degree	Nuclear	39	3	1st child (age 8)/ 1.5 years 2nd child (age 6)/2 years
P12	35	High school	Nuclear	37	4	1st child (age 5)/2 years
P13	17	Primary School	Extended	36	1	–
P14	38	Primary School	Extended	38	3	1st child (age 19)/1 year 2nd child (age 4)/6 months
P15	37	Doctorate	Nuclear	37	2	1st child (age 6,5)/23 months
P16	27	Literate	Nuclear	38	4	1st child (age 3)/10 months
P17	27	Primary School	Extended	37	1	–

### Theme 1: Breastfeeding and Life

Theme 1, “Breastfeeding and life,” focuses on how pregnant women viewed the breastfeeding process, including their feelings, thoughts, and anticipated effects on their daily lives.

#### Sub-Theme 1: Feelings about the Breastfeeding Process

The pregnant women talked about their feelings, thoughts, and experiences regarding the breastfeeding process, and almost all the participants stated that they had positive thoughts and perceptions about breastfeeding. Some primiparous pregnant women stated that they had difficulty in describing what they would experience during breastfeeding because it was a feeling they did not experience.

It’s my first pregnancy; I’m considering breastfeeding as much as I can. Because I have never experienced it, but it feels like this, I think I have experienced the feeling of motherhood, different and beautiful feelings, sacred feelings. The greatest way of motherhood is to breastfeed your child; breastfeeding is a very good feeling. (P2).

The most beautiful thing in the world is the most amazing feeling between mother and child. When you breastfeed, you experience different feelings, your love increases, you become more sensitive, and you think that you are sufficient because you breastfeed. (P4).

Two pregnant women expressed their feelings with fearful sentences because they had not experienced this feeling before.

I am worried that I will experience this for the first time, I have fears whether I will be able to breastfeed, I have fears if my milk will be enough for my baby in the future. (P1).

The thing I fear most until now is breastfeeding… (P8)

#### Sub-Theme 2: Factors Influencing the Breastfeeding Decision

It has been found that most pregnant women are conscious of the benefits of breastfeeding, and their motivation for breastfeeding is high. In fact, their immediate environment and prior experiences would not affect their determination to breastfeed.

*“My mother said that there is nothing as valuable as breast milk, in the first months, they said it was a medicine. They said it was good for more children. They told me to never give up. They told me to breastfeed my child.*

… I was self-motivated and succeeded by my own belief. (P15)

Many participants mentioned positive influence from relatives such as mothers, sisters, mothers-in-law, or aunts, as well as from their partners. These supportive individuals helped shape a more favorable attitude toward breastfeeding. However, almost none of the participants had completed any formal training or received professional support on breastfeeding.

First of all, we experienced a process with my husband for a month; we were going to start parenting education there, it is already explained there, starting from there, we already supported breast milk, and our elder relatives also supported it, starting from there we started to look at breast milk more positively, as parents, my best supporter is my husband because I think the spouse is more careful, being a perfect family starts with your spouse… (P3).

Some pregnant women stated that they feared that their breastfeeding process might be painful and challenging due to the difficulties and approaches of the people around them during the breastfeeding process.

My sister went through a very painful process for a week, so it didn’t go well for her. That’s why I’m afraid of it anyway… (P8)

For example, my aunt could not breastfeed her child, and her child could not develop either, but when I see women who are breastfeeding, their children are very healthy, some of them could not breastfeed at all. Such things can happen, it can be difficult… (P13)

Several participants noted a lack of support and even discouraging attitudes from their environment. Negative remarks could undermine their confidence, as described by one participant:

They were saying that the milk was not coming, I breastfed despite it, I did not give up… It can be very deterrent for families in that way… (P12)

People judged me, like, can’t you breastfeed? I’ve breastfed this much, I’ve breastfed that much… no one has supported me… I’m already depressed, you hit the bottom, even more, this time, you don’t breastfeed at all, or your milk runs out, you don’t feed it at all. If only we could tell these old people… (P15)

#### Sub-Theme 3: Being a Lactating Body

Primiparous pregnant women, in particular, stated that they experienced uncertainty and fears about the changes that may occur in their bodies during the breastfeeding process, and some even stated that they had prejudices in this regard.

Will I be able to recover to my previous form after giving birth? Will I not recover? I fear about it too. Breastfeeding is a good thing, what will it be like to breastfeed, will my breasts be deformed or will my breasts sag? (P1)

Now I’m going to be a first-time mom, it’s going to be weird for a baby to suck on my breast, will it change my body? I don’t know because I’ve never experienced it, so it’s going to be weird, what can I say. (P9)

On the other hand, multiparous pregnant women seem to have a more positive attitude toward the changes that may occur physically during breastfeeding.

There may be some changes in your body, you can be uncomfortable, but it didn’t bother me visually, I recovered quickly, and I didn’t experience anything negative physically. (P7)

Even if I gain weight, the health of my baby is the most important. Even if we gain weight, I can lose it in the future. The most important thing is the health of my baby… (P14)

#### Sub-Theme 4: Being a Family

A significant portion of the pregnant women expressed that they thought that being in the breastfeeding process would not make a significant change in their family life, especially in their private and social lives with their spouse, and that positive support from the family, especially from the spouse, would contribute to a more comfortable and productive process.

I think it will not cause any problems in terms of family relations, I believe my wife will support me, family support is very important in this regard. (P5)

It had a positive impact on our family life; I did not experience any negativity because I was breastfeeding. (P7)

Primiparous pregnant women, on the other hand, stated that they generally do not have a clear idea about the impacts of breastfeeding on their family life. However, they are worried about what they might experience.

Will there be a change in family structure? Will my husband’s view of me change? Probably every woman has these fears… I don’t know my husband’s reaction either, of course he has some fear, I don’t know what I will experience in reality. (P1).

It can create positive or negative effects, it can affect both ways as your life will change in general; It affects my life positively in terms of the emotions it will add to my life, and negatively in terms of time. (P2).

#### Sub-Theme 5. Breastfeeding as a Metaphor

When participants were asked to describe their feelings about imagining themselves breastfeeding in a single word, they responded with terms such as happiness, peace, joy, magnificence, miracle, and ‘the most beautiful thing in the world.

I think most things in life are those one can sacrifice for. (P3)

The smell of my baby, especially when they put her on your chest after birth, they say it is the smell of heaven, you really feel it and at that moment you forget all the pain. (P14).

### Theme 2: Spiritual Life and Breastfeeding

Under this theme, pregnant women defined the effects of spirituality on their lives and evaluated the possible effects of spirituality on breastfeeding.

#### Sub-Theme 1: Traces of Spirituality on Life

In this part, pregnant women explained the impact of spirituality on their lives. Half of the participants felt that they approached life mostly through logic, while the other half said they were guided more by their emotions and beliefs.

I’m a person focused on my beliefs, so I don’t know how it would be emotionally, but I’m more of a person focused on my beliefs. (P3)

My feelings are always a priority for me, I am a very emotional person anyway, it normally stays the same, it does not affect my spirituality. (P4)

#### Sub-Theme 2: Spirituality and Breastfeeding

In this section, pregnant women explained the impact of spirituality on breastfeeding. A significant portion of the pregnant women stated that their spirituality had no influence on their breastfeeding decisions.

I think I’ve been influenced by my spirituality in my life. I think breastfeeding feels different; it feels different than giving birth. (P6)

Spirituality is important with regard to breastfeeding, but you don’t go for it with that motivation. I’m not very impressed; I just want my baby to benefit, be healthy, and be productive. (P12).

Five of the pregnant women stated that they believed that their spiritual feelings might have an effect on their breastfeeding life.

I’m usually a naturally religious person; breastfeeding makes me happier, so negativities etc. don’t affect me much, and I still try to breastfeed. (P2)

I think that I will be more sensitive and emotional spiritually. In other words, with the state of my beliefs, motherhood requires sacrifice. (P3)

### Theme 3: Cultural Synthesis

Under this theme, pregnant women were asked to assess their opinions about breastfeeding in society from their own and other women’s perspectives. As a result, their views on breastfeeding in society were discussed.

#### Sub-Theme1: Breastfeeding in Public

Nearly all participants felt that Turkish society’s view of breastfeeding is generally positive. However, three participants noted that while society tends to be supportive, certain prejudiced or unsympathetic attitudes can still surface. Almost all participants agreed that breastfeeding in public should be discreet. Many stated that they would feel uncomfortable if someone was present while they were breastfeeding and that they would prefer to cover themselves to maintain privacy.

I think it is not convenient to breastfeed anywhere in society, the mother is a human, the baby is a human too, if there is a cover, of course, when I see a woman who breastfeeds, of course, motherhood is brought to the fore, there is nothing I can say negatively, there are always stumbling blocks. … (P3).

Sometimes I surprise my sister; she breastfeeds in every environment, but I think I can’t do it. I can’t say anything without trying. If I had to breastfeed, I would express my milk and put it in a bottle, and in an environment where women were present, I would throw a blanket over me. (P7).

They support a breastfeeding woman in society, and I think they even find it more strange when you don’t breastfeed. (P17)

## Discussion

Spirituality during pregnancy, childbirth, and the postpartum period has been confirmed in various studies (Backes et al., 2022). Almost all participants in this study had positive perceptions of breastfeeding. Spousal support is often crucial for breastfeeding mothers, and the partner’s positive attitude can strengthen a woman’s intention and prolong the breastfeeding period (Hunter & Cattelona, 2014). In certain cultures, peer support groups have proven effective in reducing social isolation after childbirth and easing the transition to motherhood (Bonia et al., 2013). A wide range of factors, including behavioral and cultural components, may influence the decision to initiate and continue breastfeeding.

Our findings indicate that body image issues may arise during pregnancy, especially during the second trimester or mid-third trimester. Although a minority of women experience dissatisfaction with their body image, such feelings can significantly affect maternal mental health and infant well-being (Przybyła-Basista et al., 2020). One study in Taiwan revealed that women with positive pre-pregnancy body image were more likely to plan to breastfeed (Larson et al., 2003). Another study suggests that mothers currently breastfeeding tend to feel more positive about their body image than those who are not (Gillen et al., 2021). Positive body image thus helps protect maternal mental health and supports adjustment to pregnancy.

Spirituality is a dimension of human existence that often becomes more pronounced in stressful or critical periods. In this study, half participants reported living life guided by logic, while the other half followed emotions and beliefs. Heidari et al. (2015) found that women with stronger spirituality were concerned about how their actions might affect their unborn children’s souls, feeling responsible for their behavior.

Muslim mothers may believe that breastfeeding is a divine gift, blessed by Allah and that they will be rewarded spiritually or have their sins forgiven during this period. Breastfeeding is considered sacred in many Islamic regions, where moral traits are believed to transfer through breast milk (Bensaid, 2021; Moran & Gilad, 2007; Uecker et al., 2016). Thus, religious faith can directly influence infant feeding practices, viewed as a form of worship. In the USA, research has shown that religion significantly influences breastfeeding among Conservative Protestants, Christians, and Muslims (Burdette & Pilkauskas, 2012). Cultural beliefs about breastfeeding vary widely and may affect breastfeeding behavior and duration. For instance, in some South and West African cultures, a new mother may remain at her maternal home for at least three months to avoid sexual relations with her spouse, thereby preventing “contamination” of her milk (Chakona, 2020). Although spirituality in Turkish society may not be fully understood, women’s awareness of the benefits of breastfeeding suggests that logic rather than spirituality often guides their decisions.

Respect for mothers’ privacy while breastfeeding is universally important and is heightened by religious beliefs in Muslim communities (Bensaid, 2021; Shaikh & Ahmed, 2006). In Islam, women must cover their bodies in the presence of non-related men, which can make public breastfeeding uncomfortable. A study by Mohamad et al. (2013) showed that Malay Muslim women’s understanding of Islamic teachings on modesty discouraged public breastfeeding. Similarly, Williamson and Sacranie (2012) found comparable attitudes among British Muslim mothers. Such findings highlight the importance for healthcare professionals to understand these concerns, especially when working with Muslim mothers.

Studies indicate that negative societal attitudes toward public breastfeeding are not limited to Muslim communities; they appear in various religious and cultural contexts. For example, in The Gambia, cultural norms do not require women to cover their breasts while breastfeeding in public, and women often dress in loose clothing when out (Chakona, 2020). However, in countries like Sweden, some women encounter negative reactions or are told to cover up, indicating a societal barrier to breastfeeding (Bonia et al., 2013; Rojjanasrirat et al., 2012). Similarly, a U.S.-based study found that women who feel uncomfortable breastfeeding in public are less likely to do so (Stuebe & Bonuck, 2011). These findings suggest that public breastfeeding is often a sensitive topic across cultures, and religious rituals and spirituality, combined with modern healthcare, can shape breastfeeding behaviors. By respecting cultural and religious differences, healthcare providers can support breastfeeding as a meaningful, nurturing, and even meditative practice (Farhadi, 2020).

### Limitations

Since the findings of this qualitative study are based on a specific setting and group, they cannot be generalized. Another limitation is that data were collected from a single hospital. It is recommended that similar studies be repeated in more diverse cultural and socioeconomic contexts to produce more comprehensive insights.

## Conclusions

Spirituality can have a significant impact on a woman's physical, emotional, and psychological well-being during pregnancy, thereby supporting a healthier breastfeeding experience. A woman’s spiritual beliefs, values, and support systems can contribute positively to both the pregnancy and breastfeeding process. Considering the role of spirituality in fostering and developing breastfeeding during pregnancy, these findings can help nurses and healthcare professionals recognize the spiritual needs of pregnant women and appreciate the protective influence of spirituality. Given the role of spirituality in fostering and developing breastfeeding during pregnancy, these results may guide nurses in recognizing pregnant women’s spiritual needs and appreciating the protective influence of spirituality. Understanding these aspects may enable healthcare professionals to provide more informed, holistic, and culturally sensitive support that enhances the breastfeeding experience for mothers.

When healthcare professionals incorporate spirituality into a holistic care approach, they are not only addressing physical health but also the emotional and mental well-being of women. This type of support, which respects cultural and individual differences, helps create a care model that honors each woman’s beliefs and values. As a result, nurses and other healthcare professionals can enhance the breastfeeding experience and overall pregnancy journey, making it more meaningful, fulfilling, and sustainable.
